# 1‐Dehydro‐6‐Gingerdione Exerts Anticancer Effects on MDA‐MB‐231 Cells and in the Xenograft Mouse Model by Promoting the Ferroptosis Pathway

**DOI:** 10.1002/ptr.8331

**Published:** 2024-10-14

**Authors:** Thi Hoa My Tran, Sanjeevram Dhandapani, Samad Abdus, Yeon‐Ju Kim

**Affiliations:** ^1^ Graduate School of Biotechnology, and College of Life Science Kyung Hee University Yongin Republic of Korea

**Keywords:** 1‐dehydro‐6‐gingerdione, anticancer, breast cancer, ferroptosis signaling

## Abstract

Breast cancer (BC) is the most prevalent malignancy among women, with millions of newly diagnosed cases emerging annually. Therefore, identifying novel pharmaceuticals for therapeutic purposes is imperative. Several natural compounds and their products have demonstrated potential in the treatment of cancer. This study examined the effects of the ginger derivative 1‐dehydro‐6‐gingerdione (1‐D‐6‐G) on BC and its mechanisms of action. MTT and colony formation assays were used to check the anticancer effect of 1‐D‐6‐G. Then the anticancer mechanism of 1‐D‐6‐G was predicted using proteomics analysis. The molecular pathway was verified by qRT‐PCR and immunobloting analysis. Additionally, the anticancer properties of 1‐D‐6‐G were investigated in vivo using xenograft mice model. Finally, an in silico study was conducted to examine the interaction of 1‐D‐6‐G and pathway‐related proteins. MTT and colony formation assay results indicated that 1‐D‐6‐G has potent cytotoxic properties against BC cells. Proteomic analysis revealed that the anticancer mechanism of 1‐D‐6‐G on MDA‐MB‐231 cells is associated with the ferroptosis signaling pathway. In addition, qRT‐PCR and immunoblotting analyses revealed that the cytotoxic effects of 1‐D‐6‐G on MDA‐MB‐231 cells were associated with ferroptosis signaling induction. Our in vivo results further confirmed the in vitro findings. The administration of 1‐D‐6‐G for 14 days exhibited anticancer properties in xenograft mice by stimulating the ferroptosis pathway without causing damage to essential organs such as the liver and kidneys. Additionally, in silico results confirmed the structural stability of the molecular interaction between 1‐D‐6‐G and ferroptosis target proteins. Our findings indicate that 1‐D‐6‐G has the potential to serve as a novel therapeutic agent for inhibiting BC progression by targeting the ferroptosis pathway.

## Graphical abstract

The schematic diagram and the main underlying mechanism of 1‐Dehydro‐6‐Gingerdione‐induced cell death in MDA‐MB‐231 cells.

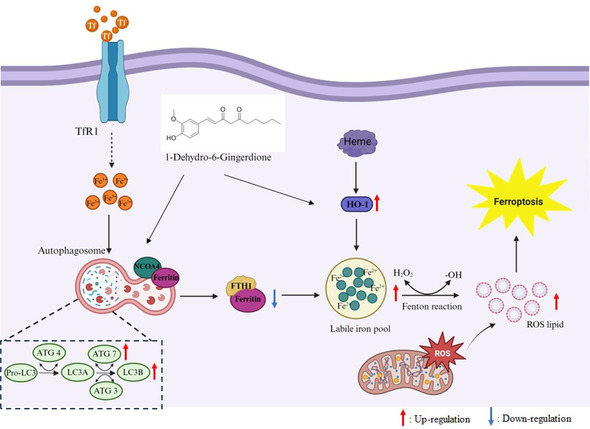

**Abbreviations**
1‐D‐6‐G: 1‐dehydro‐6‐gingerdione
AA: amino acid
A/G: albumin/globulin
ALT: alanine aminotransferase
AST: aspartate aminotransferase
ATG7: autophagy‐related 7
BC: breast cancer
BUN: blood urea nitrogen
Cis: cisplatin
Crea: creatinine
DEPs: differentially expressed proteins
FBS: fetal bovine serum
FTH1: ferritin heavy chain 1
GLU: glucose
GO: Gene Ontology
H&E: hematoxylin–eosin
HO‐1: heme oxygenase 1
IHC: immunohistochemical
KEGG: Kyoto Encyclopedia of Genes and Genomes
LC3B: microtubule‐associated proteins 1A/1B light chain 3B
MTT: 3‐(4,5‐dimethylthiazol‐2‐yl)‐ 2,5‐diphenyltetrazolium bromide
PBS: phosphate‐buffered solution
PBST: phosphate‐buffered solution with 0.5% Tween 20
PDB: Protein Data Bank
qRT‐PCR: quantitative real‐time polymerase chain reaction
RMSD: root mean square deviation
RMSF: root mean square fluctuation
ROS: reactive oxygen species
RPMI‐1640: Roswell Park Memorial Institute‐1640 medium
T‐Chol: total cholesterol
TG: total triglyceride
TP: total protein

## 1 Introduction

Breast cancer (BC) represents a significant threat to women's health globally, with the number of cases continuously rising every year (Anastasiadi et al. 2017). Recently, various treatment approaches have been established for BC, including chemopreventive agents, chemotherapeutics, and surgery. However, remission is often associated with undesirable side effects (Gurunathan et al. 2013). Therefore, it is crucial to develop cancer treatment agents with high efficacy and minimal toxicity. Currently, around 50% of all anticancer drugs are derived from natural sources, such as polyphenols, flavonoids, alkaloids, and sesquiterpenes (Mohan Shankar et al. 2022). These natural compounds have the ability to induce cell death by targeting a variety of cell signaling pathways molecules, including inflammatory cytokines, growth factors, tumor cell survival factors, and protein kinases (Haque, Brazeau, and Amin 2021). Natural products and their primary bioactive compounds have been reported to be effective in the prevention and treatment of BC (Y. Li et al. 2017). Thus, the utilization of natural products in the development of innovative anticancer drugs appears to be a promising strategy for addressing BC treatment challenges.

Ginger (*Zingiber officinale* Roscoe), belonging to the Zingiberaceae family, has been used as a flavor enhancer (spice) or medicinal herb since ancient times and remains widely used even today (Kiyama 2020). Ginger has demonstrated considerable efficacy in treating neurodegenerative diseases, migraines, diabetes, respiratory disorders, and cardiovascular disease (Akinyemi et al. 2015; Ho, Chang, and Lin 2013; F. Y. Huang et al. 2019; Mao et al. 2019; White 2007). More than 115 and 63 different compounds have been identified in dried and fresh ginger, respectively. These compounds include shogaol and gingerol derivatives (such as 1‐dehydro‐10‐gingerdione, 1‐dehydro‐6‐gingerdione, 10‐shogaol, 6‐shogaol, 10‐gingerol, 8‐gingerol, and 6‐gingerol), which are considered bioactive components (Kota, Krishna, and Polasa 2008; Simon et al. 2020). Ginger compounds have been reported to improve antioxidant levels and reduce damage in the kidneys and liver of rats (Kota, Krishna, and Polasa 2008). The anticancer properties of these compounds have been demonstrated extensively in cancer research (R. Wang et al. 2023). In particular, the cytotoxic effects of 6‐gingerol have been investigated using several cancer cell lines (de Lima et al. 2018). Additionally, 6‐shogaol has been confirmed to exhibit anticancer properties against various types of cancers, including breast, colon, lung, and pancreatic (Kou et al. 2018). Furthermore, 1‐dehydro‐6‐gingerdione (1‐D‐6‐G) has been reported to markedly inhibit inflammation in macrophages (F. Li et al. 2012); chronic inflammation can lead to several types of cancer (Mantovani et al. 2008). Moreover, numerous studies have investigated the biological features of ginger‐derived compounds; however, research on 1‐D‐6‐G and its potential, especially its anticancer effects, remains limited.

Therefore, this study aimed to investigate the anticancer effects of 1‐D‐6‐G on BC cells and xenograft mice and its mechanism of action against cancer. Our findings indicated that 1‐D‐6‐G suppresses BC by inducing ferroptosis. To the best of our comprehension, this is the initial research to examine the potential anticancer potential of 1‐D‐6‐G. The results of this study will pave the way for more in‐depth investigations into the potential therapeutic applications of 1‐D‐6‐G.

## 2 Materials and Methods

### 2.1 Materials

1‐D‐6‐G was obtained from the Natural Product Institute of Science and Technology (Anseong, Korea). MDA‐MB‐231 cells were purchased from the Korean Cell Line Bank (Seoul, Korea). Roswell Park Memorial Institute‐1640 medium (RPMI‐1640), penicillin–streptomycin, and fetal bovine serum (FBS) were purchased from GenDEPOT (USA). Antibodies against heme oxygenase 1 (HO‐1, ab52947), autophagy‐related 7 (ATG7, MA5‐35317), microtubule‐associated proteins 1A/1B light chain 3B (LC3B, ab48394), ferritin heavy chain 1 (FTH1, PA5‐27500), and β‐actin (sc‐47778) were purchased from Abcam and Thermo Fisher. Primers used in our research were designed and supplied by Macrogen (Songdo, Korea). The primer sequences are presented in Table S1.

### 2.2 Cell Culture and Viability Assay

MDA‐MB‐231 cells were cultured in RPMI‐1640 containing 10% FBS and 1% penicillin–streptomycin at 37°C with 5% CO_2_. To assess cytotoxicity, MDA‐MB‐231 cells were placed at a density of 2 × 10^5^ cells per well in 96‐well plates. After 24 h, cells received treatments with various concentrations (20, 40, 60, 80, and 100 μM) of 1‐D‐6‐G and cisplatin (Cis) with 50 μM, while the control group cells were only treated with the free medium. Following 24 h, the cytotoxicity was evaluated using the (3‐(4,5‐dimethylthiazol‐2‐yl)‐2,5‐diphenyltetrazolium bromide) (MTT, M6494) reduction assay, as previously described (Mi, Choi, et al. 2022).

### 2.3 Colony Formation Assay

MDA‐MB‐231 cells were placed at 2 × 10^5^ cells/mL density for 24 h and then treated with 1‐D‐6‐G and Cis for the colony formation assay. After 24 h, the cells were stained with 0.5% crystal violet (Invitrogen) and subsequently washed five times using PBS. The quantification of stained colonies was determined using the ImageJ software (MD, USA).

### 2.4 Proteomics Analysis

MDA‐MB‐231 cells were placed in a cell culture dish and stabilized until reaching 80% confluence. The cells were washed with PBS and then 80 μM of 1‐D‐6‐G was added. The control group got only free medium. After 24 h, the cells were harvested using Pierce RIPA Buffer (Thermo Fisher Scientific) that contained the protease inhibitor cocktail (GenDEPOT) and subsequently vortexed every 15 min for 1 h. The lysate was centrifuged at 13500 rpm for 20 min at 4°C to collect the supernatant and the concentration of protein was determined using the Bradford assay. Proteomics analysis was performed as described previously (Xu et al. 2022).

### 2.5 Quantitative Real‐Time Polymerase Chain Reaction (qRT‐PCR)

MDA‐MB‐231 cells were seeded into a 60 mm dish at a density of 2 × 10^5^ cells/mL for 24 h. The cells were treated with our samples and then incubated for 24 h. The total RNA of the cells was extracted using the TRIzol reagent kit (Invitrogen, CA, USA). The AmfiRivert cDNA Synthesis Platinum Enzyme Mix (GenDEPOT) was used to reverse‐transcribe total RNA into cDNA. qRT‐PCR was conducted using the AmfiSure qGreen Q‐PCR Master Mix (GenDEPOT).

### 2.6 Western Blot

MDA‐MB‐231 cells were seeded into a 60 mm dish at a density of 2 × 10^5^ cells/mL for 24 h. The cells were treated with our samples and then incubated for 24 h. After that, the cells were harvested using Pierce RIPA Buffer (89900, Thermo Fisher Scientific) containing a protease inhibitor cocktail (P3200‐001, GenDEPOT) and then vortexed every 15 min for 1 h. The lysate was centrifuged at 13,500 rpm for 20 min at 4°C for collecting the supernatant as crude protein. The total protein was loaded into 10% SDS‐PAGE gel and transferred to a PVDF membrane (W7031‐270, Thermo Fisher Scientific). The membranes have been in 5% skim milk for 2 h at room temperature and then kept in primary antibodies overnight at 4°C. After washing three times with PBST (PBS with 0.5% Tween 20), the membranes were further kept in the secondary antibody for 1 h at room temperature. The West‐Q Pico ECL Solution (W3652‐E1, GenDEPOT) was used for protein visualization. The quantities of each protein band were calculated using ImageJ.

### 2.7 Malondialdehyde (MDA), Reactive Oxygen Species (ROS), and Iron Assay

MDA‐MB‐231 cells were seeded into a 60‐mm plate at a density of 2 × 10^5^ cells/mL. After 24 h, the cells were treated with our samples. The cells were collected and then lysated with BHT solution after 24 h treatment. The lysate was centrifuged at 13,000*g* for 10 min to collect the supernatant. Then lipid peroxidation was identified by measuring the level of MDA using a Lipid Peroxidation Assay Kit (ab118970, Abcam, UK) following the manufacturer's instructions.

MDA‐MB‐231 cells were seeded into a 96‐well plate at a density of 2 × 10^5^ cells/mL for 24 h. One hundred microliters of ROS Red Working Solution was added into each well of the cell plate and cultured in an incubator at 37°C/5% CO_2_. After 1 h, the cells were treated with our samples and kept in an incubator for 30 min. The ROS level was measured using a fluorescent microplate reader (SpectraMax ABS plus; CA, USA). The production of ROS was quantified using the cell ROS analysis kit (ab186027, Abcam, UK), following the manufacturer's protocol.

MDA‐MB‐231 cells were seeded into a 60‐mm plate at a density of 2 × 10^5^ cells/mL for 24 h. The cells were treated with our samples for 24 h. The cells were collected with ice‐cold PBS after 24 h treatment. The cell solution was centrifuged at 1000*g* for 5 min to collect the pellet. The pellet was gently sonicated on ice and then centrifuged at 16,000*g* for 10 min at 4°C to collect the supernatant for the assay. Finally, ferrous iron levels were determined using an Iron Colorimetric Assay Kit (ab83366, Abcam, UK), following the manufacturer's guidance.

### 2.8 In Vivo Xenograft Model and Experimental Schedule

Female BALB/c Nude mice (4 weeks, 18–20 g) were purchased from Saerombio Inc., (Uiwang, Korea) and kept in a controlled room with food and water ad libitum. All experimental procedures were carried out according to the Principles of Laboratory Animal Care (NIH publication #80‐23, revised in 1996) and the Kyung Hee University Animal Care and Use Guidelines (KHGASP‐20‐375). After 1 week of adaptation, MDA‐MB‐231 cells (2 × 10^8^) were subcutaneously injected into the left back of the mice. When the volume of the tumor reached approximately 100 mm^3^, the mice were randomly separated into five groups (*n* = 5): control tumor (ConT), 1‐D‐6‐G groups included 2, 4, 8, and 5 mg/kg 5‐FU (F6627). Another group, referred to as the control (Con) group comprised five mice without tumor induction. The mice were orally given 1‐D‐6‐G and 5‐FU once daily for a duration of 14 days. The Con and ConT groups were orally administered PBS. The tumor volume and body weight of all mice were measured daily. Following the completion of the experiment, all the mice were sacrificed. The kidney, liver, tumor tissues, and blood were isolated and weighed for further experiments.

### 2.9 Serum Biochemical Analysis

The collected blood was centrifuged for 10 min at 2000 rpm to obtain the serum, which was subsequently utilized for biochemical analysis. A Fuji Dri‐Chem analyzer (3500, Fuji Photo Film Co., Osaka, Japan) was employed to determine various biomarkers, including albumin, aspartate aminotransferase (AST), alanine aminotransferase (ALT), the levels of albumin/globulin (A/G), glucose (GLU), total cholesterol (T‐Chol), total triglyceride (TG), creatinine (Crea), and total protein (TP).

### 2.10 Hematoxylin–Eosin (H&E) and Immunohistochemical (IHC) Staining

According to a previous study (Xu et al. 2017), tumor tissues were fixed with 10% formalin and then embedded in paraffin. After the paraffin hardened, tissues were sectioned and subsequently stained with H&E for microscopic observation or subjected to IHC staining, which was conducted using a mouse and rabbit‐specific HRP/DAB (ABC) detection IHC kit (ab 64264). A microscope was used to observe the tissues, and the detection of specific proteins in the tissues was identified by a brown–yellow color.

### 2.11 In Silico Analysis

#### 2.11.1 Ferroptosis Proteins Preparation

The RCSB Protein Data Bank (PDB) was used to derive the 3D crystal structures of HO‐1 (1N3U), ATG7 (AF‐095352‐F1), LC3B (3VTU), and FTH1 (5N26) proteins (Berman et al. 2000). The protein crystal structure was cleaned by removing co‐crystallized ligands, other chains, H atoms, and the solvent. The Protein Preparation Workflow in Schrödinger 2023‐3 was performed to optimize the protein structure with correct bond ordering, insertion of hydrogen atoms and missing residues, and removal of the water molecule from the protein, as the protein structures obtained from the PDB were unsuitable for molecular docking and further investigation (Samad, Huq, and Rahman 2022). The OPLS4 force field was used for protein preparation (Z. Li et al. 2023).

#### 2.11.2 1‐D‐6‐G Compound Processing

The PubChem database was used to obtain the 3D structures of erastin (control ligand) and 1‐D‐6‐G in SDF format (Kim et al. 2021). The LigPrep feature of Maestro v13.7 was used to prepare the ligand. Finally, using the Epik module, the OPLS4 force field was applied to optimize the ligand structure (Pokhrel et al. 2021).

#### 2.11.3 Molecular Docking of Ferroptosis Proteins With 1‐D‐6‐G Compound

Molecular docking was performed using the Glide package (OPLS4 force field) of the Schrödinger 2023‐3 software. The target proteins and ligands were investigated together, and the binding site of the receptors was chosen using the SiteMap package, Schrödinger 2023‐3. A receptor grid box (*X* = 10, *Y* = 10, *Z* = 10) was constructed using the Glide package with default parameters. The binding score and their interactions were exhibited using the Maestro viewer (Samad, Huq, and Rahman 2022).

#### 2.11.4 Molecular Dynamic (MD) Simulation and Post‐Dynamic MM‐GBSA

MD simulations can validate the stability of ligand‐protein complexes in a particular and synthetic environment (Ahammad et al. 2021). The MD simulation results are described using root mean square deviations (RMSD) and root mean square fluctuation (RMSF). To assess the structural integrity of the protein complex, the Desmond module of Schrödinger 2023‐3 analyzed the protein‐binding stability of the selected ligands, including their target proteins. We performed simulations (200 ns) with the OPLS4 force field, maintaining a pH of 7.4 for enhanced binding complex identification (Ahammad et al. 2021). To provide a simulation environment, we initially solvated the protein complex with water molecules and subsequently enclosed it in an orthorhombic box to establish the complex boundaries. To ensure a salt concentration of 0.15 M, Na^+^ and Cl^−^ ions were neutralized. The simulation was performed with a recording interval of 200 ps at 1.01325 bar and 310 K (Tang et al. 2023). The SID module of the Schrödinger package was used to analyze the dynamic simulation quality. The RMSD and RMSF were assessed using simulated trajectories.

### 2.12 Statistical Analysis

The results were reported as the mean standard deviation. For statistically significant differences between the two groups, the Student's *t*‐test was used, and the data were deemed significant at *p* < 0.05, *p* < 0.01, and *p* < 0.001.

## 3 Results

### 3.1 Anticancer Effect of 1‐D‐6‐G in HCC‐38 and MDA‐MB‐231 Cells

**FIGURE 1:**
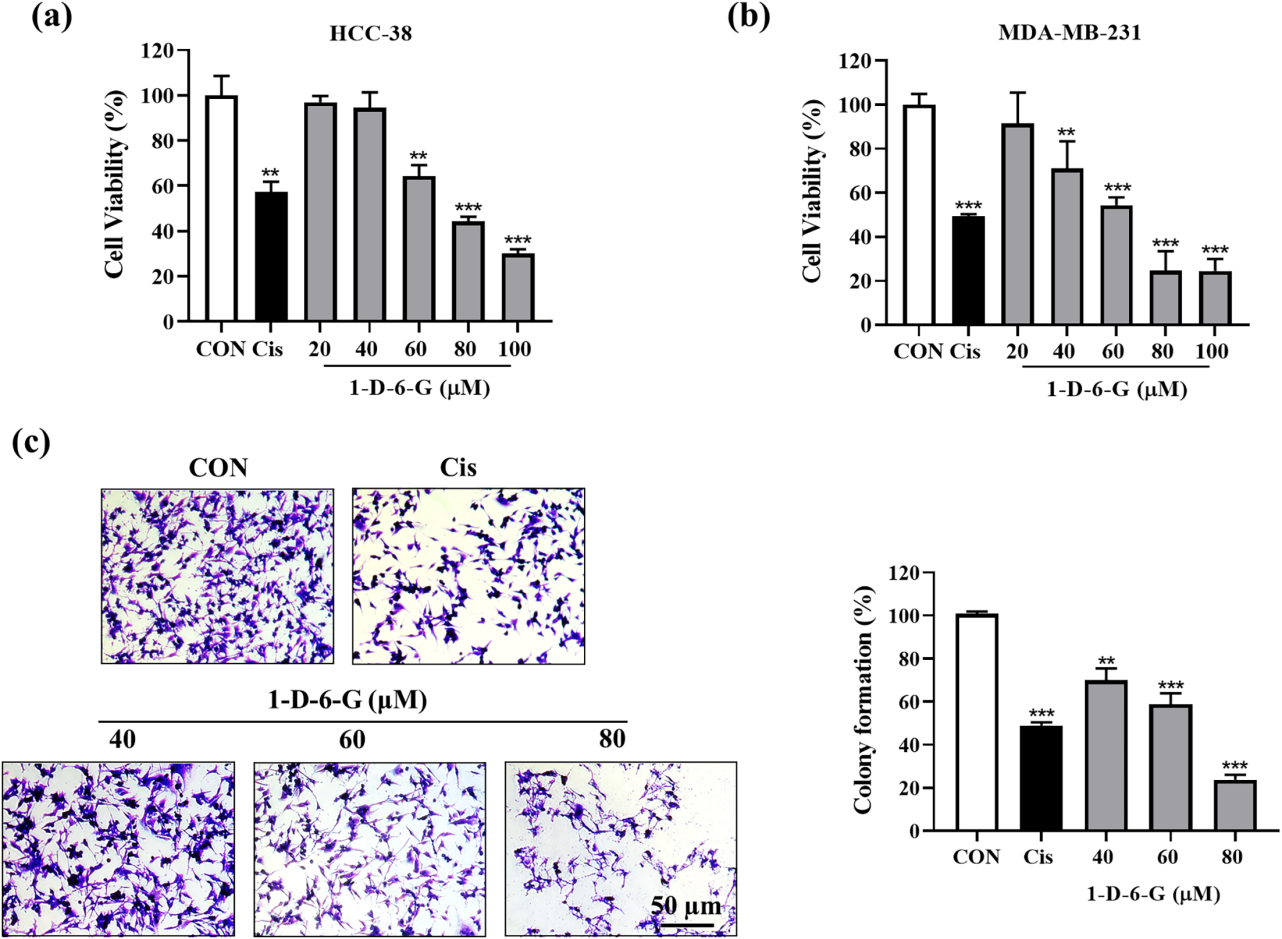
Cytotoxic effects of 1‐D‐6‐G on HCC‐38 and MDA‐MB‐231 cells. (a) Cell viability of HCC‐38 cell. (b) Cell viability of HCC‐38 cell MDA‐MB‐231 cell. (c) Colony formation assay and data evaluation. Results are presented as mean ± SD (*n* = 3). Significance is presented as **p < 0.01 and ***p < 0.001 versus Con.

MTT and colony formation assays were utilized to evaluate the cytotoxicity effect of 1‐D‐6‐G on HCC‐38 and MDA‐MB‐231 cells. As shown in Figure 1a, 1‐D‐6‐G reduced the viability of HCC‐38 cells from 96.94% to 30.13% at concentrations from 20 to 100 μM. Based on the results in Figure 1b, we determined the cytotoxic impact of 1‐D‐6‐G on MDA‐MB‐231 cells within a dose range of 20–100 μM. 1‐D‐6‐G decreased the cell viability from 91.47% to 24.5% in a dose‐dependent manner and the half maximum inhibitory concentration (IC_50_) value of 1‐D‐6‐G was 71.13 μM. As expected, Cis (50 μM), a positive control, exerted a substantial toxic effect (49.25%) against MDA‐MB‐231 cells. In comparison, the inhibition effect of 1‐D‐6‐G on MDA‐MB‐231 cells is better than that on HCC‐38 cells. Therefore, a colony formation assay was performed to assess the effect of 1‐D‐6‐G on MDA‐MB‐231 cancer cell proliferation (Figure 1c). Cells in the control group exhibited well‐defined morphological structures and formed several colonies. In contrast, 1‐D‐6‐G remarkably suppressed MDA‐MB‐231 colony formation by 70.02%–23.77% in a dose‐dependent manner. Specifically, the inhibitory effect of 1‐D‐6‐G (80 μM) on colony formation was greater than that of Cis. These results illustrated that 1‐D‐6‐G exerts a significant cytotoxic effect on MDA‐MB‐231 cells.

### 3.2 Effect of 1‐D‐6‐G on Protein Expressions in MDA‐MB‐231 Cells

**FIGURE 2:**
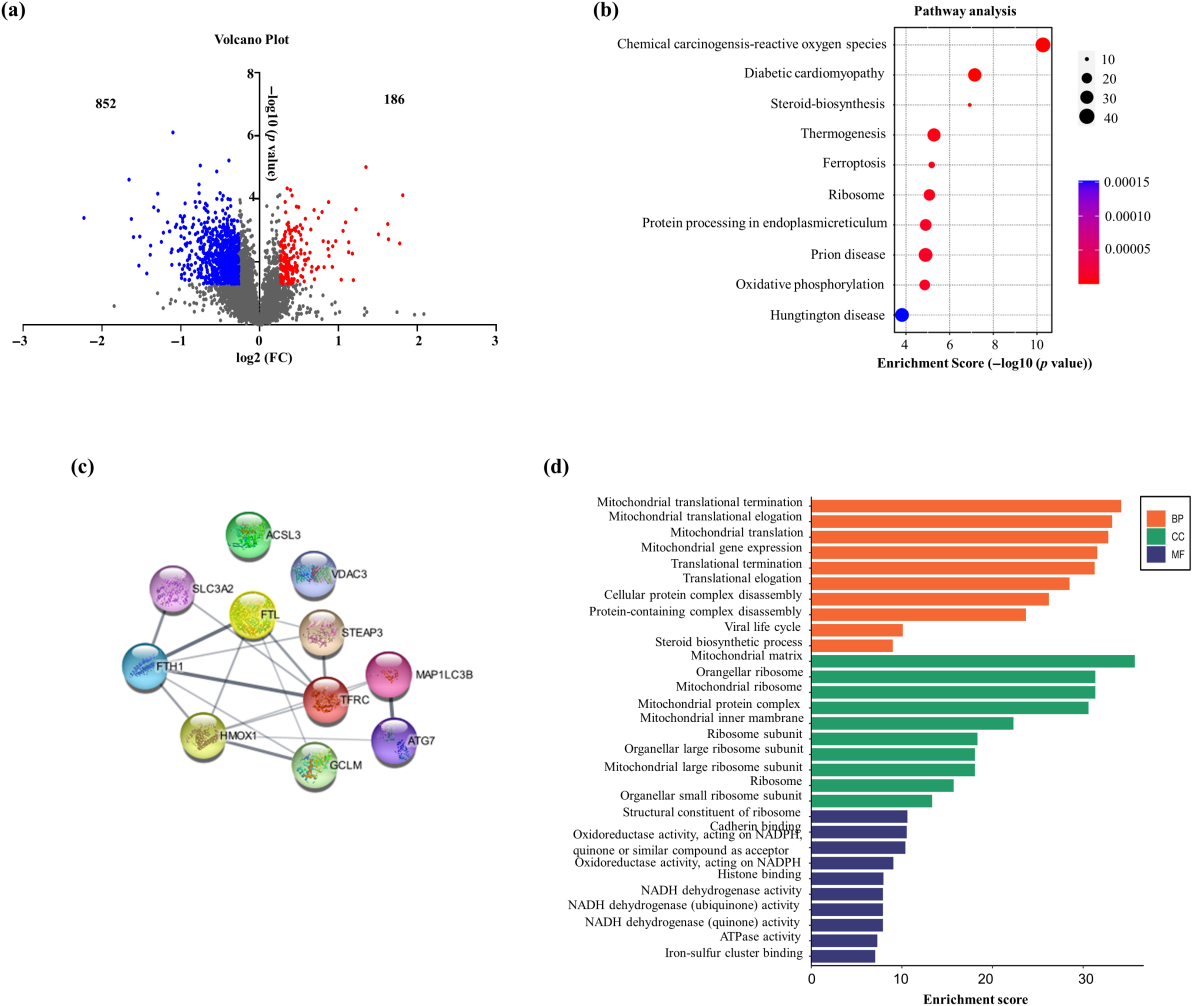
Identification of proteins and analysis of pathway enrichment. (a) Genes with differential expressions displayed using volcano plot and (b) Kyoto Encyclopedia of Genes and Genomes (KEGG) pathway analysis. (c) STRING displayed the interactions between proteins which involved in ferroptosis signaling. (d) Protein ontology includes biological process (BP), cellular component (CC), and molecular interaction (MF).

To elucidate the mechanism underlying the inhibitory effects of 1‐D‐6‐G on MDA‐MB‐231 cells, we performed proteomics analysis to identify the protein–protein interactions that may contribute to cancer cell death. Quantitative proteomic analysis of 1‐D‐6‐G‐treated MDA‐MB‐231 cells revealed the presence of numerous differentially expressed proteins (DEPs) (Figure 2). Volcano plot, DEPs data, and Kyoto Encyclopedia of Genes and Genomes (KEGG) enrichment pathway analyses were used to investigate the distribution of significantly expressed proteins. As shown in Figure 2a, the volcano plot displays DEPs 852, which were downregulated shown in blue and 186 were upregulated indicated in red. Scatterplots of KEGG enrichment for biological processes are shown in Figure 2b. The expression of the chemical carcinogenesis–ROS signaling was the most pronounced pathway. The diabetic cardiomyopathy pathway was also strongly expressed. ROS are known for their significant involvement in cell death processes, such as apoptosis, ferroptosis, and autophagy (Endale, Tesfaye, and Mengstie 2023). Diabetic cardiomyopathy treatment targets Ferroptosis‐induced cell death through the regulation of iron hemostasis and lipid peroxidation (X. Wang et al. 2022). Interestingly, ferroptosis is a key signaling pathway in 1‐D‐6‐G‐inhibited MDA‐MB‐231 cells. Therefore, 1‐D‐6‐G may inhibit cancer cell growth by activating the Ferroptosis signaling pathway. Figures S1 and 2c show the ferroptosis signaling pathway and associated protein, including HO‐1, ATG7, LC3B, FTH1, which were expressed in 1‐D‐6‐G‐treated MDA‐MB‐231 cells. HO‐1 performs its role in Ferroptosis at the cellular iron level and generates ROS (Chiang, Chen, and Chang 2019). It is reported that ferroptosis is a type of autophagy‐dependent cell death (Q. Huang et al. 2022). Depletion of autophagy‐related genes, including LC3B and ATG7, prevents ferroptotic cancer cell death (J. Li et al. 2021). FTH1 is a component of ferritin that can limit ferroptosis (Hou et al. 2016).

In addition, gene ontology (GO) and pathway enrichment analyses were performed to assess the biological functions of all the identified DEPs. GO comprised molecular interactions, biological processes, and cellular components (Figure 2d). The result revealed that the majority of regulatory protein expression in 1‐D‐6‐G‐treated MDA‐MB‐231 cells was mostly related to mitochondrial activities. Mitochondria participate in the execution of several types of regulated cell death, including extrinsic, intrinsic apoptosis and autophagy (Mattson, Gleichmann, and Cheng 2008; Xie et al. 2018). Interestingly, induction of Ferroptosis has been demonstrated to result in mitochondrial ROS production, mitochondrial fragmentation, and loss of the mitochondrial membrane potential; therefore, mitochondria play an important role in the ferroptosis process (Bebber et al. 2020). Therefore, we hypothesized that ferroptosis is a crucial signaling pathway in the cell death induced by 1‐D‐6‐G. Along these lines, we investigated ferroptosis‐associated indicators in 1‐D‐6‐G‐treated cells in order to understand the molecular mechanisms underlying 1‐D‐6‐G‐induced cell death in MDA‐MB‐231.

### 3.3 Effect of 1‐D‐6‐G on the Ferroptosis Signaling Pathway

**FIGURE 3:**
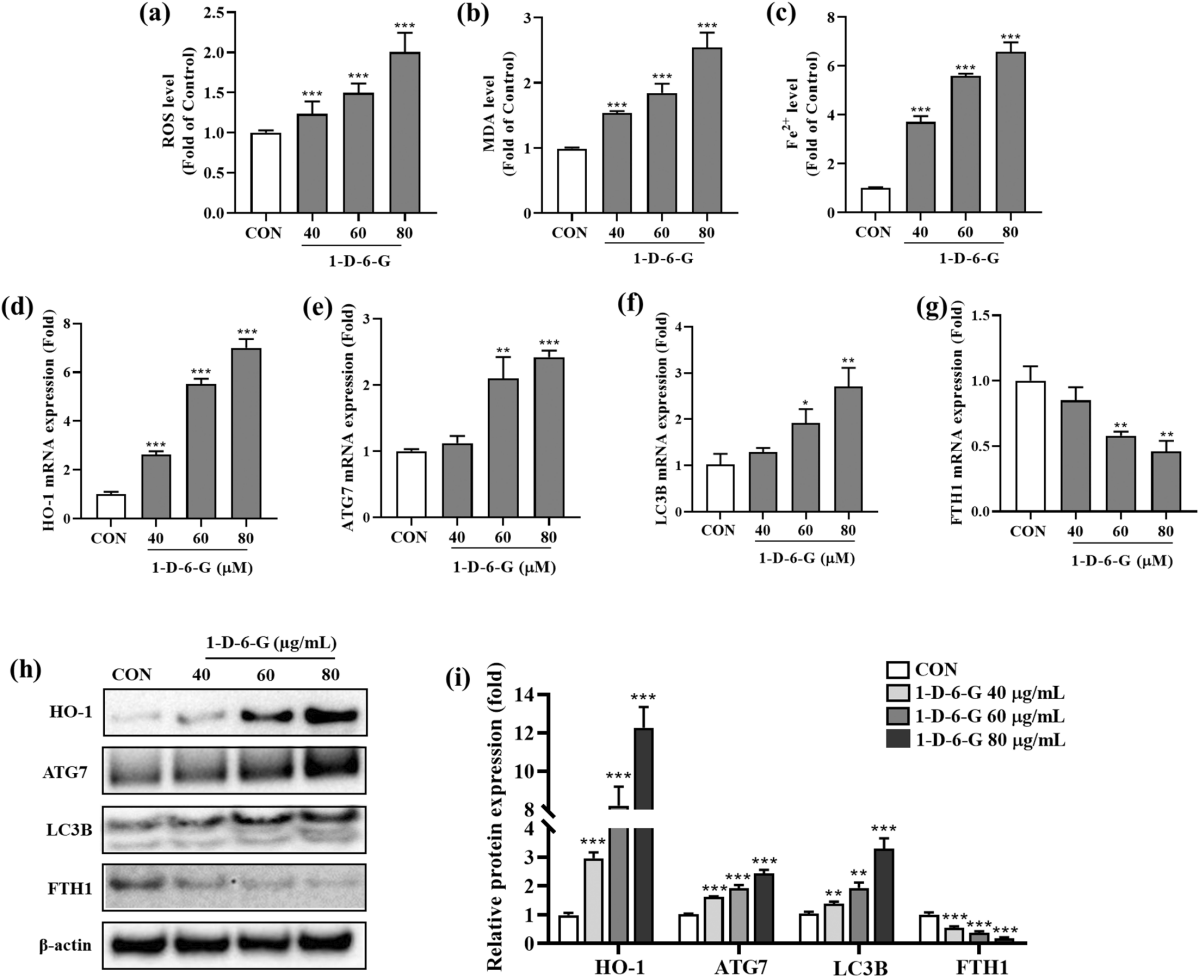
1‐D‐6‐G induced ferroptosis signaling in MDA‐MB‐231 cells. (a) ROS production, (b) MDA production, (c) Fe^2+^ production, (d–g) gene expression of HO‐1, ATG7, LC3B, and FTH1, (h) protein expression of HO‐1, ATG7, LC3B, and FTH1, (i) quantitative analysis of HO‐1, ATG7, LC3B, and FTH1 normalized to β‐actin. Results are presented as the mean ± SD (*n* = 3). Significance is presented as *p < 0.05, **p < 0.01, and ***p < 0.001 versus Con.

Ferroptosis is a form of ROS‐dependent cell death characterized by two major biochemical features: lipid peroxidation and iron accumulation (Tang et al. 2021). Thus, we investigated whether the cytotoxicity of 1‐D‐6‐G was related to oxidative stress, lipid peroxidation, and iron accumulation in MDA‐MB‐231 cells. Figure 3a showed that 1‐D‐6‐G concentration‐dependently increased the ROS levels (1.2‐, 1.5‐, and 2.0‐fold, respectively) in MDA‐MB‐231 cells. In addition, the treatment of BC cells with 1‐D‐6‐G significantly increased MDA levels (1.5‐, 1.8‐, and 2.5‐fold, respectively) (Figure 3b), which is a key lipid peroxidation marker (Ghonimi et al. 2021). Furthermore, treatment of MDA‐MB‐231 cells with 1‐D‐6‐G enhanced Fe^2+^ level (3.7‐, 5.6‐, and 6.6‐fold, respectively) (Figure 3c). Our data suggested that 1‐D‐6‐G can induce ferroptosis by promoting the ROS, MDA, and Fe^2+^ levels in MDA‐MB‐231 cells.

Based on these proteomics results, key proteins such as HO‐1, ATG7, LC3B, and FTH1 were selected to examine the mechanism of the ferroptosis pathway (Lee et al. 2020). Furthermore, qRT‐PCR and western blot analyses were performed to determine whether the expression levels of these crucial proteins were correlated with the ferroptosis signaling pathway described above. As shown in Figure 3d–f, the expression of genes related to the Ferroptosis signaling pathway, including *HO‐1*, *ATG7*, and *LC3B*, was substantially upregulated in 1‐D‐6‐G‐treated MDA‐MB‐231 cells. Moreover, the expression of the *FTH1* gene was considerably suppressed in these cells (Figure 3g). Similarly, 1‐D‐6‐G substantially increased the protein levels of HO‐1, ATG7, and LC3B while decreasing the protein expression of FTH1 (Figure 3h,i). These findings indicate that 1‐D‐6‐G treatment induces MDA‐MB‐231 cell cytotoxicity by suppressing the expression of genes and proteins associated with the ferroptosis signaling pathway.

Ferroptosis is related to profound alterations in the morphology of the mitochondria (H. Wang et al. 2020). In Figure 3k, the cells in the control group had normal tubular mitochondrial morphology. In contrast, 1‐D‐6‐G induced mitochondria clusters and increased membrane density. Those mitochondrial clusters exhibited decreased mitochondrial function, suggesting mitochondrial damage. Previous studies have shown that changes in mitochondrial morphology during ferroptosis are associated with increased membrane density (Y. Liu et al. 2023). These results partly contribute to the study that 1‐D‐6‐G induced MDA‐MB‐231 cell death through promoting ferroptosis.

### 3.4 Effect of 1‐D‐6‐G and Ferrostatin‐1 on the Ferroptosis Signaling Pathway

**FIGURE 4:**
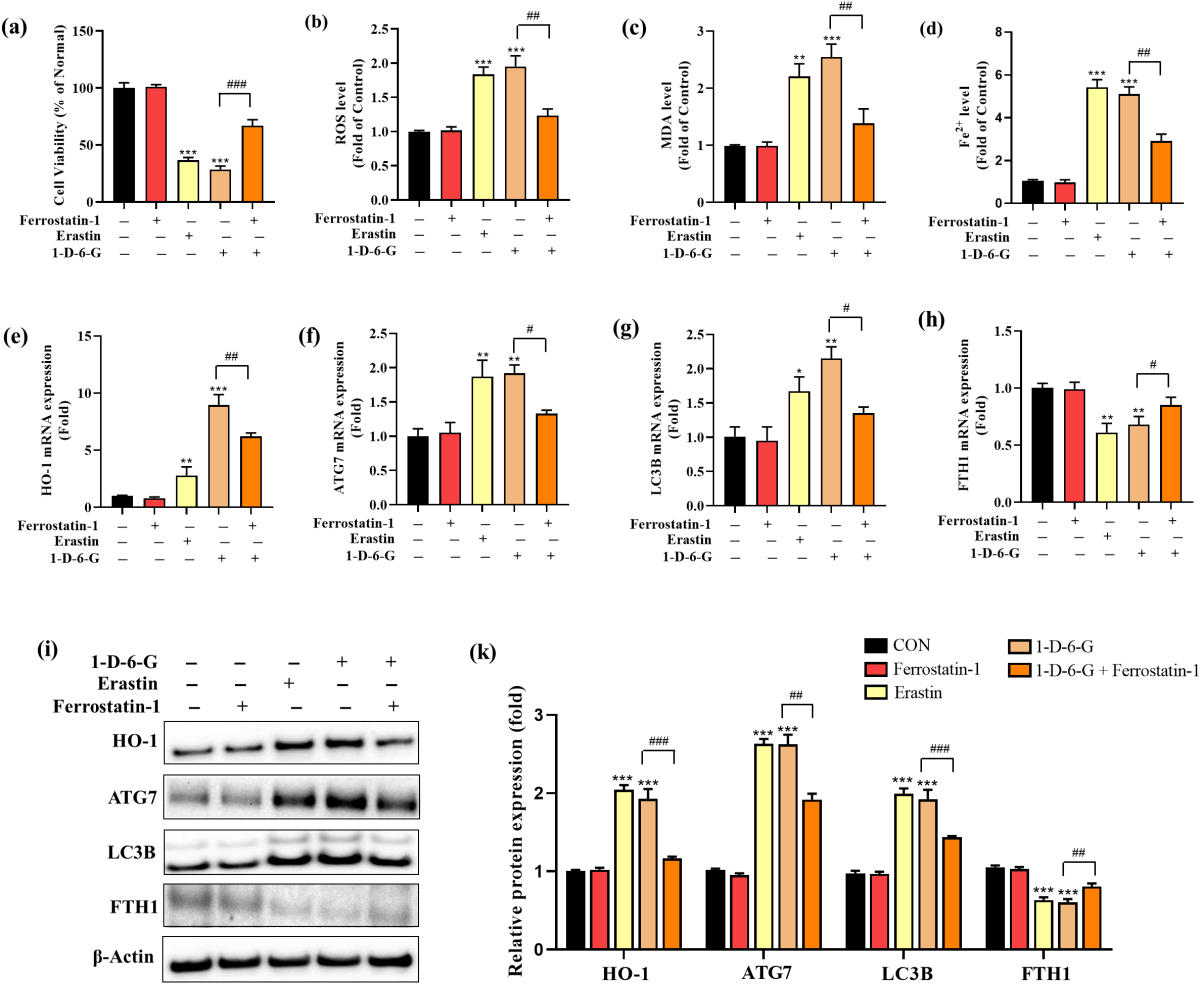
Effect of inhibitor or inducer in ferroptosis signaling in 1‐D‐6‐G‐treated MDA‐MB‐231 cells. (a) Cell viability, (b) ROS production, (c) MDA production, (d) Fe^2+^ production, (e–h) Gene expression of HO‐1, ATG7, LC3B, and FTH1 (i) protein expression of HO‐1, ATG7, LC3B, and FTH1, (k) quantitative analysis of HO‐1, ATG7, LC3B, and FTH1 normalized to β‐actin (*n* = 3). Results are presented as the mean ± SD (*n* = 3). Significance is presented as *p < 0.05, **p < 0.01, and ***p < 0.001 versus Con and ^#^ *p* < 0.05, ^##^ *p* < 0.01, and ^###^ *p* < 0.001 versus 1‐D‐6‐G‐treated group.

To verify the role of 1‐D‐6‐G in inducing ferroptosis in MDA‐MB‐231 cells, ferrostatin‐1, a widely recognized ferroptosis inhibitor, was employed to block 1‐D‐6‐G‐induced ferroptosis. Erastin, a well‐known ferroptosis inducer, was used as the positive control. As shown in Figure 4a, the MTT assay demonstrated that 1‐D‐6‐G treatment markedly reduced cell viability compared to the non‐treated group; however, this reduction was significantly recovered when MDA‐MB‐231 cells were co‐treated with 1‐D‐6‐G and ferrostatin‐1 (2.0 μM). Similarly, the erastin group exhibited significantly decreased cell viability compared with the control group. Next, we assessed the 1‐D‐6‐G‐induced changes in ROS, lipid peroxidation, and iron levels in MDA‐MB‐231 cells. 1‐D‐6‐G significantly increased ROS, MDA, and intracellular Fe^2+^ production, which are considered to be related to ferroptosis sensitivity (Figure 4b–d). Conversely, the elevated levels of ROS, MDA, and iron caused by 1‐D‐6‐G were significantly reduced in the presence of ferrostatin‐1 in MDA‐MB‐231 cells. These findings demonstrate that cell death caused by 1‐D‐6‐G may be prevented by inhibiting ferroptosis in MDA‐MB‐231 cells.

Subsequently, the effect of 1‐D‐6‐G on gene and protein expressions in MDA‐MB‐231 cells treated with ferrostatin‐1 was evaluated using qRT‐PCR and western blot analyses. As shown in Figure 4e–g, the mRNA expression levels of HO‐1, ATG7, and LC3B in the 1‐D‐6‐G treatment group were significantly increased, whereas ferrostatin‐1 co‐treatment with 1‐D‐6‐G significantly decreased. Furthermore, the suppression of FTH1 mRNA expression caused by 1‐D‐6‐G treatment was reversed by ferrostatin‐1 and 1‐D‐6‐G co‐treatment (Figure 4h). Consistent with the qRT‐PCR data, 1‐D‐6‐G elevated HO‐1, ATG7, and LC3B protein expression and inhibited FTH1 protein expression. However, ferrostatin‐1 dramatically reversed the 1‐D‐6‐G effects (Figure 4i,k). These results confirm that 1‐D‐6‐G has the potential to trigger ferroptosis in MDA‐MB‐231 cells by regulating the gene and protein expression of HO‐1, ATG7, LC3B, and FTH1.

### 3.5 Effect of 1‐D‐6‐G on Promoting Oxidative and Iron‐Dependent Cell Death

**FIGURE 5:**
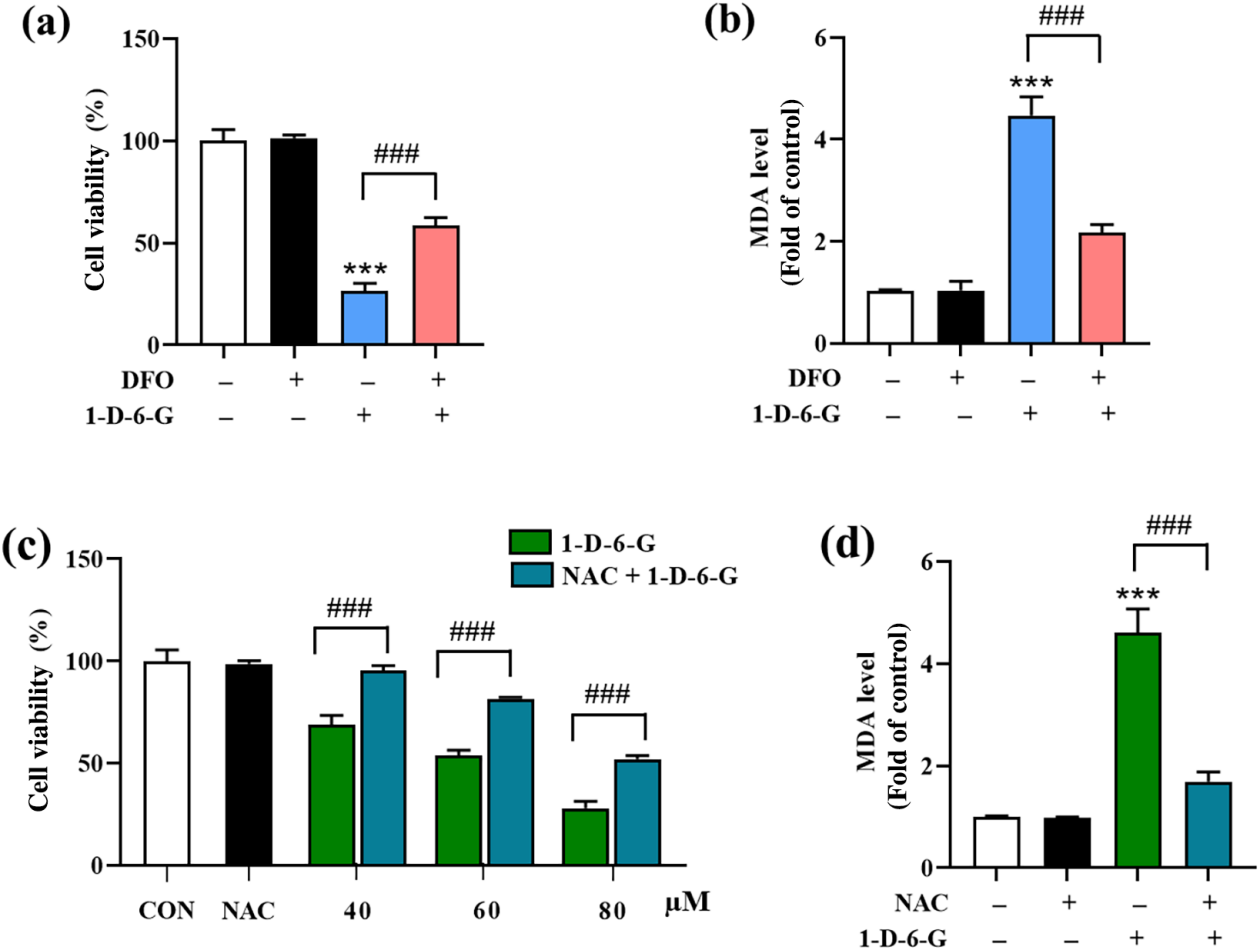
1‐D‐6‐G induces iron‐dependent and oxidative cell death. MDA‐MB‐231 cells were co‐treated with 1‐D‐6‐G (80 μM) and DFO (100 μM) or NAC (5 mM). (a) Cell viability of 1‐D‐6‐G and DFO co‐treatment, (b) MDA levels of 1‐D‐6‐G and DFO co‐treatment, (c) cell viability of 1‐D‐6‐G and NAC co‐treatment, (d) MDA content 1‐D‐6‐G and DFO co‐treatment. Data are expressed as the mean ± S.D (*n* = 3). ***p < 0.001 versus control group; ^##^ *p* < 0.01, ^###^ *p* < 0.001 versus 1‐D‐6‐G‐treated group.

Deferoxamine (DFO) is a widely used iron chelator that binds to ferrous ions, effectively preventing ferroptosis in conditions of iron overload (Dixon et al. 2012). Therefore, DFO was employed to investigate the role of iron in the ferroptosis signaling pathway mediated by 1‐D‐6‐G, which results in the death of cancer cells (Figure 5a,b). The results showed that 1‐D‐6‐G significantly induced cell death and lipid peroxidation in the MDA‐MB‐231 cells. However, these effects were reversed when the cells were co‐treated with DFO (100 μM), suggesting that iron plays a critical role in 1‐D‐6‐G‐induced cell death by promoting the ferroptosis pathway.

Additionally, to explore the potential role of elevated ROS levels in the cytotoxicity of 1‐D‐6‐G, we used N‐acetyl‐l‐cysteine (NAC) as an inhibitor of ROS. MDA‐MB‐231 cells were treated with a combination of NAC (5 mM) and 1‐D‐6‐G at concentrations of 40, 60, and 80 μM. As shown in Figure 5c, NAC co‐treatment dramatically reduced the cytotoxicity effect of 1‐D‐6‐G on MDA‐MB‐231 cells. In addition, the presence of NAC also attenuated the increase in MDA level induced by 1‐D‐6‐G (Figure 5d). These results suggested that 1‐D‐6‐G induces BC cell death by enhancing ROS to promote the ferroptosis pathway.

### 3.6 Effects of 1‐D‐6‐G in Xenograft Mouse Model Implanted With MDA‐MB‐231 Cells

Natural products have traditionally been considered promising anticancer candidates, and several single compounds have shown substantial anticancer activity in vitro (Atanasov et al. 2021). Nevertheless, in vitro studies are inadequate for evaluating the potential anticancer properties of certain compounds. Thus, thymus‐deficient nude mice bearing MDA‐MB‐231 xenografts were used to investigate the anticancer efficacy of 1‐D‐6‐G. The positive control utilized in the experiment was 5‐FU (5 mg/kg).

**FIGURE 6:**
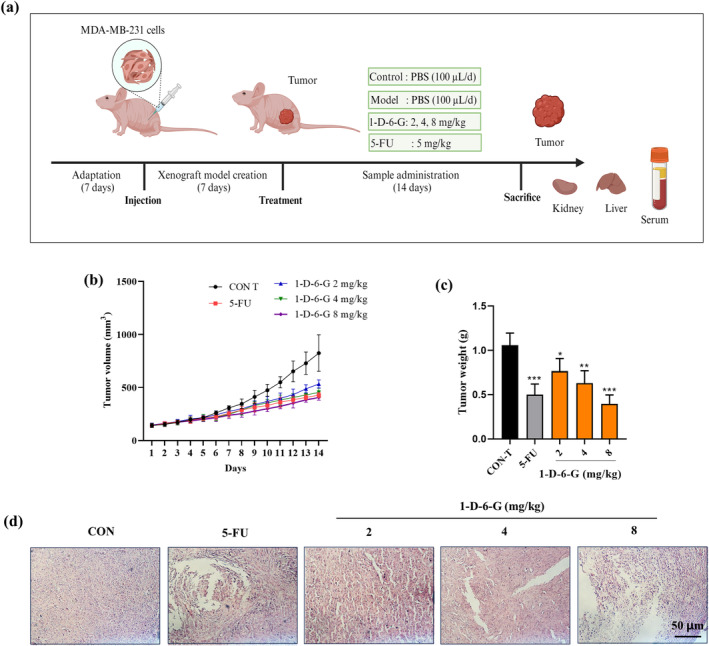
1‐D‐6‐G administration suppressed tumor growth in MDA‐MB‐231 xenograft‐bearing mice. (a) Design of in vivo experiments, (b) tumor volume, (c) tumor weight, (d) H&E staining of tumor. Results are presented as the mean ± SD (*n* = 5). Significance is presented as *p < 0.05, **p < 0.01, and ***p < 0.001 versus Con‐T.

As depicted in Figure 6a, each group of mice was orally administered various doses of 1‐D‐6‐G and 5‐FU after establishing a xenograft model implanted with MDA‐MB‐231 cells. During dosing, the tumor volume of the mice in each group was measured to determine the suppressive effect of 1‐D‐6‐G on tumor development. As predicted, 5‐FU significantly inhibited tumor growth compared with the control (Figure 6b). Additionally, daily 1‐D‐6‐G treatment at concentrations of 2, 4, and 8 mg/kg resulted in a significant reduction in tumor size compared with only saline administration (tumor control). As shown in Figure 6c, 1‐D‐6‐G administration resulted in a dose‐dependent reduction in tumor weight in the xenograft mouse model. Notably, 1‐D‐6‐G at a dose of 8 mg/kg had an even greater impact on decreasing tumor weight and size in mice than the 5‐FU treatment. Furthermore, H&E staining was used to evaluate the effect of 1‐D‐6‐G on tumor structure. As shown in Figure 6d, the 1‐D‐6‐G‐treated groups displayed significant nuclear rupture, tumor cell shrinkage, cavity formation, and necrosis of the tumor tissue. These results suggested that 1‐D‐6‐G effectively caused tumor tissue damage and tumor cell death.

**FIGURE 7:**
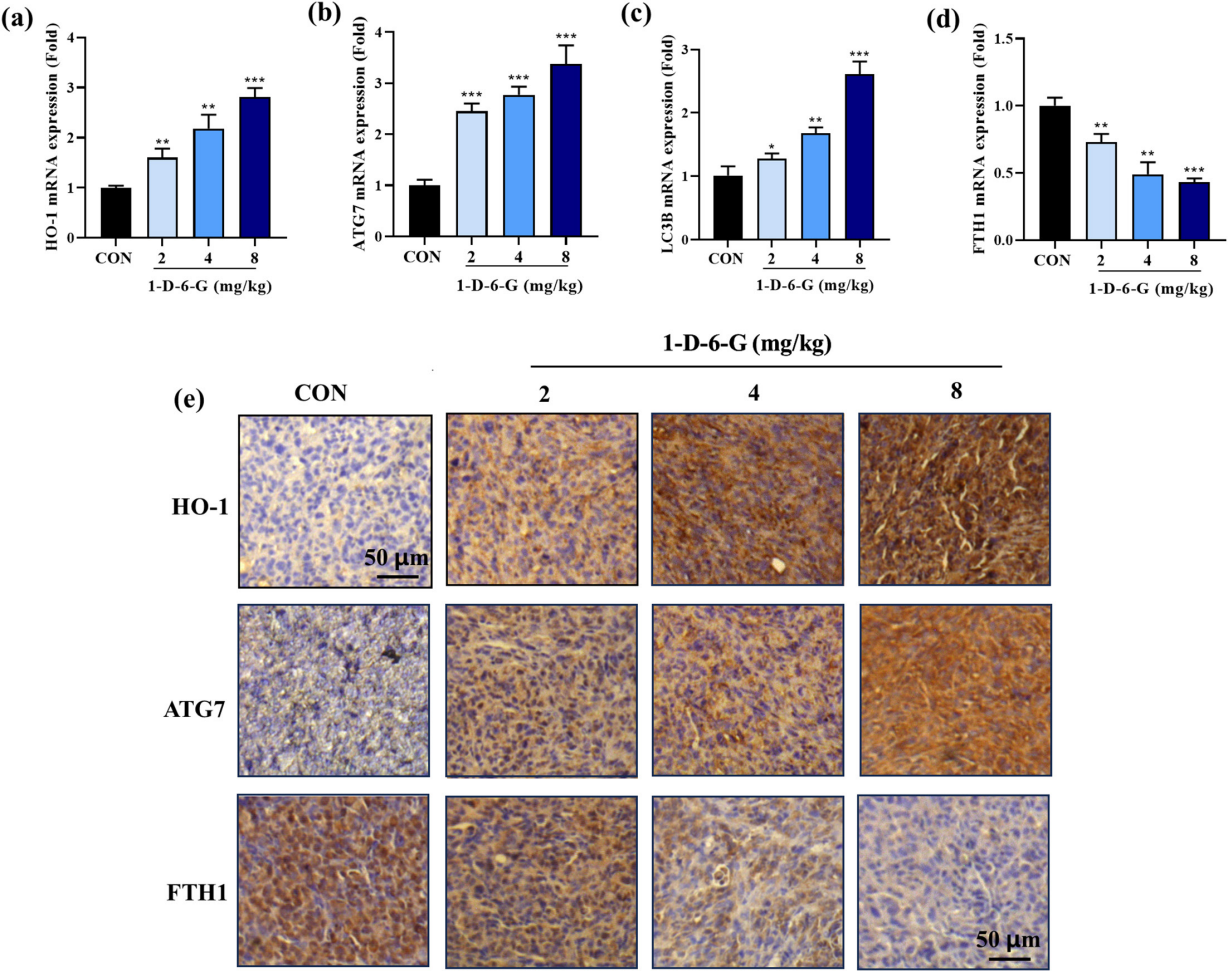
1‐D‐6‐G administration induced the ferroptosis signaling pathway to inhibit tumor growth in MDA‐MB‐231 xenograft‐bearing mice. (a–d) Gene expression of HO‐1, ATG7, LC3B, and FTH1, (e) IHC staining of HO‐1, ATG7, and FTH1 proteins in tumor tissues. Results are presented as the mean ± SD (*n* = 3). Significance is presented as *p < 0.05, **p < 0.01, and ***p < 0.001 versus Con.

Further experiments were conducted to evaluate the expression of genes correlated with the ferroptosis pathway in tumor tissues to validate their involvement in tumor inhibition (Figure 7a–d). The mRNA levels of HO‐1, ATG7, and LC3B were increased in the tumor tissues of 1‐D‐6‐G‐treated xenograft mice than in Con‐T mice. However, mRNA expressions of FTH1 in tumor tissues were significantly alleviated in 1‐D‐6‐G‐administered xenograft mice in a dose‐dependent manner. Consistent with the above findings, IHC staining also showed that the densities of HO‐1 and ATG7 were dramatically increased, whereas the density of FTH1 was markedly reduced in the tumor tissues of the 1‐D‐6‐G‐treated group (Figure 7e). These results demonstrate that 1‐D‐6‐G exhibits its anticancer properties by inducing the ferroptosis signaling pathway.

### 3.7 Effect of 1‐D‐6‐G on In Vivo Cytotoxicity

**FIGURE 8:**
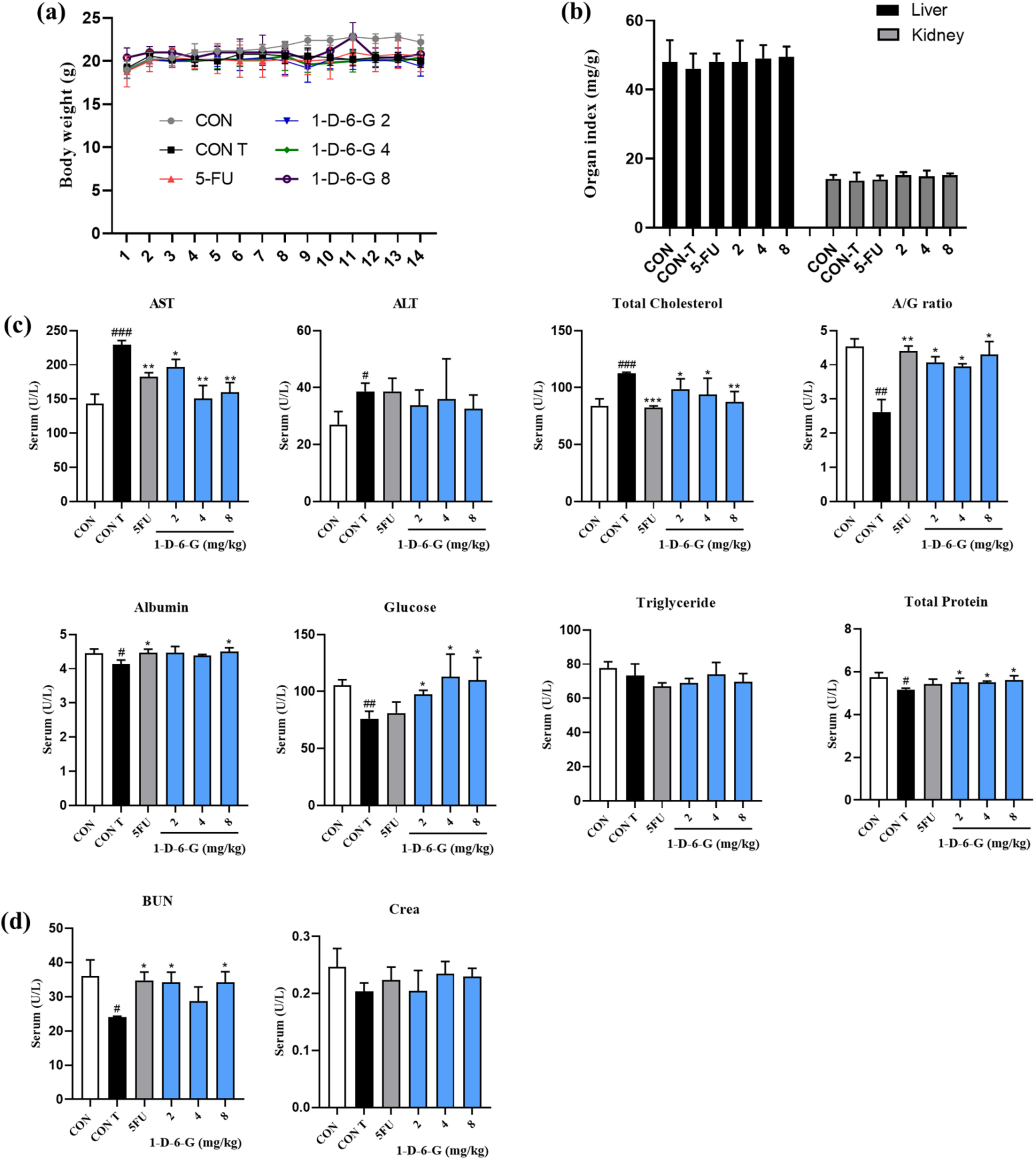
In vivo toxic effects of 1‐D‐6‐G during the treatment period. (a) Body weight, (b) liver and kidney index, (c) detection of the liver‐relative markers AST, ALT, and total cholesterol, A/G ratio, albumin, glucose, triglyceride, total protein, (d) detection of kidney relative markers BUN and Crea. Results are presented as the mean ± SD (*n* = 3). Significance is presented as *p < 0.05, **p < 0.01, and ***p < 0.001 versus Con‐T; ^#^ *p* < 0.05, ^##^ *p* < 0.01, and ^###^ *p* < 0.001 versus Con.

Anticancer drugs have various side effects because they may be cytotoxic to both cancer and normal cells. Some side effects have the potential to cause serious disorders and impair organ functioning in the body (Lowenthal and Eaton 1996). Hence, it is essential to validate the safety profile of novel drugs. In this study, we checked the body weight of mice during the 1‐D‐6‐G administration period to assess the toxic effects of 1‐D‐6‐G. Figure 8a shows that mice in the control group exhibited a consistent increase in body weight. The body weights of the Con‐T, 5FU, and 1‐D‐6‐G groups showed no statistically significant difference. Because drugs are metabolized in both the liver and kidneys, it is imperative to conduct toxicological assessments of these organs. Kidney and liver toxicity was investigated by evaluating weight indices and other blood biomarkers in 1‐D‐6‐G‐administered mice. Figure 8b further illustrates that the liver and kidney indices were not substantially different between the groups. Liver damage alters the quantity of enzymes released into the bloodstream. Liver function biomarkers, namely AST, ALT, and T‐Chol, were significantly increased in the Con‐T group, suggesting that the MDA‐MB‐231‐xenograft mice had impaired liver function (Figure 8c). However, compared with Con‐T treatment, 1‐D‐6‐G treatment considerably attenuated the levels of these biomarkers. In addition, the Con‐T group had a lower A/G ratio and lower Alb, GLU, TG, and TP levels than the Con group did. In contrast, the A/G ratio and Alb, GLU, and TP contents were substantially elevated in the 1‐D‐6‐G group than in the Con‐T group; however, there was no statistically significant difference in the TG content. These results indicate that 1‐D‐6‐G is not only non‐hepatotoxic but also protects the liver from damage caused by MDA‐MB‐231 xenografts. Subsequently, the renal function of all mice, including BUN and Crea levels, was evaluated, and the results are presented in Figure 8d. The BUN levels were dramatically reduced in the Con‐T group, which was reversed by the 1‐D‐6‐G administration. Crea content was not substantially different between the Con‐T and 1‐D‐6‐G‐treated groups. These findings suggested that 1‐D‐6‐G did not have adverse effects on the kidneys and ameliorated the impairment caused by MDA‐MB‐231 xenografts.

### 3.8 Molecular Docking Analysis of 1‐D‐6‐G With Ferroptosis‐Related Targets Proteins

Recently, computational drug design techniques, including docking simulations and MD, have become the most popular and applied methods for analyzing and discovering pharmaceuticals and biologically active molecules based on specific targets (Mahmud et al. 2021; Opo et al. 2021). Molecular docking is a useful method for screening and determining the ideal intermolecular interactions between phytochemical compounds and targeted proteins (Ahammad et al. 2021). Therefore, we used computer‐aided drug design bioinformatics methods to determine the ability of 1‐D‐6‐G to induce ferroptosis.

We employed molecular docking to predict atomic‐level interactions between 1‐D‐6‐G and ferroptosis‐related proteins with erastin, a ferroptosis inducer, used as a positive control. The best docking of the receptor‐ligand interactions is presented in Table 1. The interactions among the compounds, proteins, and their structures are shown in Figure S2. The interaction between 1‐D‐6‐G and ferroptosis‐related proteins (HO‐1, ATG7, LC3B, and FTH1) had a binding energy of −4.99, −3.81, −2.89, and −4.22 kcal/mol, respectively. Interestingly, the binding energy of 1‐D‐6‐G with HO‐1, ATG7, and FTH1 proteins was lower than that of erastin. Additionally, hydrogen bonds, a form of non‐bonded interaction between ligands and receptors, were identified. We observed that the interacting H‐bonding residues of 1‐D‐6‐G had two H‐bonds with the HO‐1, ATG7, LC3B, and FTH1 proteins. In contrast, erastin showed only one H‐bonded residue with these proteins, indicating that the H‐bonding of 1‐D‐6‐G with ferroptosis‐associated proteins is stronger than that of erastin. H‐bonds play an important role in maintaining protein–ligand interactions (Chen et al. 2016). These findings suggest that 1‐D‐6‐G can efficiently target key proteins involved in ferroptosis signaling.

**TABLE 1:** Molecular docking score and H‐bonding of 1‐D‐6‐G and erastin with HO‐1, ATG7, LC3B, and FTH1.

Targeted proteins	Docking score (kcal/mol)		H‐bonding	
	1‐D‐6‐G	Erastin	1‐D‐6‐G	Erastin
HO‐1	−4.99	−4.23	Arg136, Gln38	Gly139
ATG7	−3.81	−2.78	Lys338, Asp334	Arg380
LC3B	−2.89	−4.79	Asn88, Glu40	Gly89
FTH1	−4.22	−3.88	Asn25, Asn109	Asp89

**FIGURE 9:**
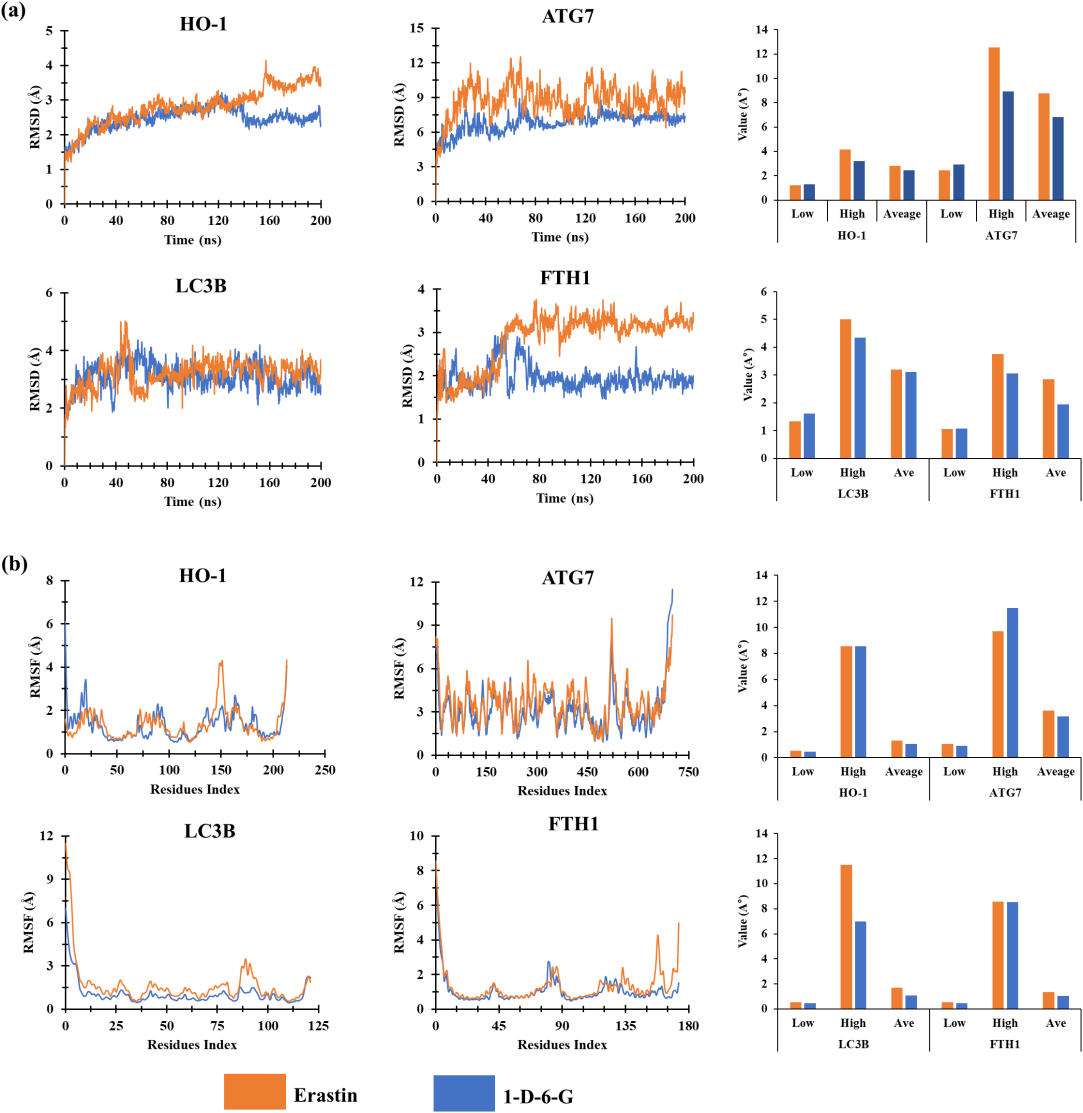
MD simulation of ligand‐proteins complex during a 200 ns simulation period. (a) RMSD values of control ligand and 1‐D‐6‐G with HO‐1, ATG7, LC3B, and FTH1, (b) RMSF values of control ligand and 1‐D‐6‐G with HO‐1, ATG7, LC3B, and FTH1.

As shown in Figure 9a, RMSDs of Cα atoms were calculated for 1‐D‐6‐G‐ and erastin–protein complexes to assess the protein structure stability. Comparing the RMSD values of HO‐1, ATG7, and LC3B proteins and 1‐D‐6‐G with those of the control ligand, stable confirmation was observed with the values 140–200, 83–200, and 70–200 ns, respectively. Consequently, FTH1 showed a small fluctuation at 158 ns, after which it remained stable from 78 to 200 ns. In the control ligand, FTH1 and HO‐1 proteins were stable after 138 and 170 ns, respectively, and the remaining proteins were unstable in the 200 ns simulation. Moreover, average RMSD values of the 1‐D‐6‐G with HO‐1 (2.459 Å), ATG7 (6.801 Å), LC3B (3.109 Å), and FTH1 (1.952 Å) proteins were lower than those of the control ligand with HO‐1 (2.813 Å), ATG7 (8.779 Å), LC3B (3.199 Å), and FTH1 (2.852 Å), suggesting that these proteins and 1‐D‐6‐G interaction is more stable than control ligand interactions.

RMSF represents fluctuations in amino acid (AA) residues in the protein structure. The RMSF values of the AA residues measure the degree of stability of the AA residues in an intricate system (Samad, Huq, and Rahman 2022). In 1‐D‐6‐G, the highest fluctuating AA was Asn30 (RMSF = 3.41 Å), Asp522 (RMSF = 7.72 Å), Val95 (RMSF = 1.49 Å), and Asp84 (RMSF = 2.66 Å) for HO‐1, ATG7, LC3B, and FTH1, respectively. In the control ligand, the highest fluctuating residue was Gly161 (RMSF = 4.29 Å), Leu523 (RMSF = 9.48 Å), Ser91 (RMSF = 3.49 Å), and Pro161 (RMSF = 4.27 Å) for HO‐1, ATG7, LC3B, and FTH1, respectively. The highest RMSF value of 1‐D‐6‐G was also lower than that of the control ligand with ferroptosis‐related proteins, indicating that 1‐D‐6‐G shows more stability than erastin during RMSF analysis (Samad, Huq, and Rahman 2022).

## 4 Discussion

Ginger is an essential ingredient for cooking, as well as a valuable herb in traditional medicine, known for its efficacy in treating various diseases, including cancer, hypertension, arthritis, and inflammation (Altman and Marcussen 2001; Mao et al. 2019). Various bioactive compounds present in ginger have been identified and evaluated; however, the potential anticancer properties of 1‐D‐6‐G have not been studied previously. The cell viability showed that 1‐D‐6‐G was cytotoxic to HCC‐38 and MDA‐MB‐231 BC cells. We observed that the inhibition of 1‐D‐6‐G on MDA‐MB‐231 cells is better than that on HCC‐38 cells with the same concentration treatment. Therefore, MDA‐MB‐231 cells were chosen for further study. This study investigated the anticancer effects of 1‐D‐6‐G on BC cells and MDA‐MB‐231 xenograft‐bearing mice. Similar to our results, Zhou et al. (2021) demonstrated that polyphyllin III can induce cell death in MDA‐MB‐231 by promoting ferroptosis signaling. Additionally, 6‐gingerol has been shown to inhibit the viability and migration of prostate cancer cells by promoting the ferroptosis pathway (C. M. Liu et al. 2022). In the present study, we confirmed the anticancer effects of 1‐D‐6‐G on BC cells.

Proteomics is a powerful technique for predicting and identifying proteins whose encoding genes are differentially expressed in tissues or cells under different conditions (Mir et al. 2023). Based on the GO and KEGG‐enriched analyses in the present study, we hypothesized that 1‐D‐6‐G toxicity in MDA‐MB‐231 cells is associated with Ferroptosis signaling. As shown in Figure 4a, treatment with ferrostatin‐1, a specific inhibitor of ferroptosis, resulted in a significant decrease in the cytotoxic effect of 1‐D‐6‐G. Furthermore, ferrostatin‐1 reduced the increased levels of ROS, MDA, and Fe^2+^ in MDA‐MB‐231 induced by 1‐D‐6‐G. Similarly, X. J. Mi, Park, et al. (2022) found that *Cirsium japonicum*‐loaded AuNPs induced Ferroptosis‐related cancer cell death by increasing ROS and Fe^2+^ levels and promoting lipid peroxidation and iron accumulation. These findings demonstrate the critical role of ferroptosis signaling in the anticancer properties of 1‐D‐6‐G in MDA‐MB‐231 cells.

Ferritin is the primary intracellular iron storage complex whose increased expression leads to the inhibition of ferroptosis (Theil 2004; Yang and Stockwell 2008). It has been suggested that ferritin‐containing autophagosomes fuse with lysosomes to generate autophagolysosomes. Ferrous iron (Fe^2+^), the reduced form of iron, is released when ferritin is digested in autophagolysosomes (Lee et al. 2020). Consequently, one of the most crucial mechanisms for preserving iron homeostasis is the autophagic degradation of ferritin. In this study, 1‐D‐6‐G considerably increased ATG7 and LC3B expression and significantly decreased FTH1 expression in MDA‐MB‐231 cells (Figure 4e–h). In addition, 1‐D‐6‐G elevated the expression of HO‐1 in MDA‐MB‐231 cells (Figure 3d), which can trigger Fe^2+^ and ROS production by interacting with physical and chemical reagents (X. J. Mi, Park, et al. 2022; Ryter et al. 2002). Numerous studies have suggested that ROS are produced via autophagic degradation of ferritin (Bresgen and Eckl 2015; Hou et al. 2016). Additionally, ferrostatin‐1 attenuated the effect of 1‐D‐6‐G in upregulating ATG7, LC3B, and HO‐1 expression and downregulating FTH1 expression in MDA‐MB‐231 cells (Figure 4d–h), confirming the degradation of ferroptosis leads to the inhibition of autophagy‐related genes and the improvement of ferritin. These results indicate that 1‐D‐6‐G induces ferroptosis in MDA‐MB‐231 cells by promoting autophagy‐related proteins, resulting in ferritin degradation. Our findings clarify the mechanism through which autophagy‐induced ferritin degradation leads to ferroptosis, which causes cancer cell death.

Furthermore, we used an in vivo model of immunosuppressed mice carrying MDA‐MB‐231 xenografts to validate our in vitro findings. As expected, tumor progression notably decreased in xenograft mice administered with 1‐D‐6‐G compared with the tumor control group (Figure 5a–d). 1‐D‐6‐G treatment (8 mg/kg) was more effective than 5‐FU treatment (5 mg/kg) in xenograft‐bearing mice. This indicates that natural compounds can be considered as potential anti‐BC drug candidates. In addition, tumor tissues obtained from 1‐D‐6‐G‐administration groups exhibited a notable increase in the levels of molecules associated with the ferroptosis signaling pathway (Figure 6). Liu et al. also found that 6‐gingerol reduces tumor growth in tumor‐bearing mice by inducing ferroptosis (Tsai, Xia, and Sun 2020). These findings support our in vitro study that the anticancer properties of 1‐D‐6‐G are mediated by ferroptosis, indicating the tumor suppression in vivo induced by 1‐D‐6‐G is, at least in part, due to ferroptosis.

Finally, an in silico virtual process was performed to evaluate the interaction between 1‐D‐6‐G and ferroptosis‐related proteins. Molecular docking results showed that 1‐D‐6‐G had greater negative binding affinities than the control ligand when interacting with HO‐1, ATG7, and FTH1 proteins. A previous study reported that ligands with higher negative binding affinities have higher stability (Samad, Huq, and Rahman 2022). The stability of a protein with ligand complexes is also verified using molecular dynamics simulation (Aljahdali, Molla, and Ahammad 2021). The RMSD values in complex systems show the stability level of the substances, and the RMSF values determine the mean fluctuation that assesses the cohesiveness of the ligand–protein interaction (Krupanidhi et al. 2020). 1‐D‐6‐G exhibited lower RMSD and RMSF values than those of the control ligand (Figure 8). Consequently, 1‐D‐6‐G and HO‐1, ATG7, LC3B, and FTH1 protein interactions showed good stability, indicating that 1‐D‐6‐G could target the key ferroptosis proteins.

The anticancer properties of 1‐D‐6‐G were confirmed for MDA‐MB‐231 BC cells, and its effective in vitro concentration range was 40–80 μM in the current study. However, the toxicity of 1‐D‐6‐G on normal breast cells in vitro was not investigated, which is limited in this study. According to Nair and Jacob (2016), the 1‐D‐6‐G dose is converted from mice to humans lowering the body surface area per unit of body weight; hence, the human equivalent dose (HED) should be divided by the mouse dose. The clinically nontoxic and effective dose of 1‐D‐6‐G is 0.56 mg/kg based on a 20 g mouse dose. Human clinical trials are necessary to evaluate 1‐D‐6‐G due to its great efficacy and lack of toxicity.

## 5 Conclusions

This study showed that 1‐D‐6‐G inhibited the proliferation of MDA‐MB‐231 cells. Using proteomic analysis, the underlying mechanism by which 1‐D‐6‐G regulates specific proteins in MDA‐MB‐231 cells was assessed. The activation of ferroptosis by 1‐D‐6‐G was demonstrated in vitro in MDA‐MB‐231 BC cells. 1‐D‐6‐G induces ferroptosis in cancer cells via ferritin degradation. Furthermore, 1‐D‐6‐G significantly inhibited tumor growth in xenograft‐bearing mice. The potential anticancer effects of 1‐D‐6‐G on BC cells may be associated with ferroptosis‐mediated cell death and tumor inhibition. According to safety tests, 1‐D‐6‐G reversed liver and kidney damage induced by MDA‐MB‐231 xenografts. These findings offer initial evidence for the potential use of 1‐D‐6‐G as a candidate for BC treatment in clinical applications.

## Author Contributions

**Thi Hoa My Tran:** data curation, writing – original draft. **Sanjeevram Dhandapani:** software. **Samad Abdus:** software. **Yeon‐Ju Kim:** conceptualization.

## Ethics Statement

All animal experiments were conducted in strict accordance with Kyung Hee University's Animal Ethics Committee guidelines and the “National animal management regulations of South Korea.”

## Conflicts of Interest

The authors declare no conflicts of interest.

## Supporting information


Data S1.

## Data Availability

All data generated or analyzed during this study are included in this published article and its additional information.
