# Supplementary material for: 1‐Dehydro‐6‐Gingerdione Exerts Anticancer Effects on MDA‐MB‐231 Cells and in the Xenograft Mouse Model by Promoting the Ferroptosis Pathway
Source: Phytother Res. 2024 Oct 14;38(12):5901–17. doi: 10.1002/ptr.8331 (PMC11634822; doi:10.1002/ptr.8331)
Supplement: Supplementary file 1 — Data S1. [file PTR-38-5901-s001.docx]

Supplementary data


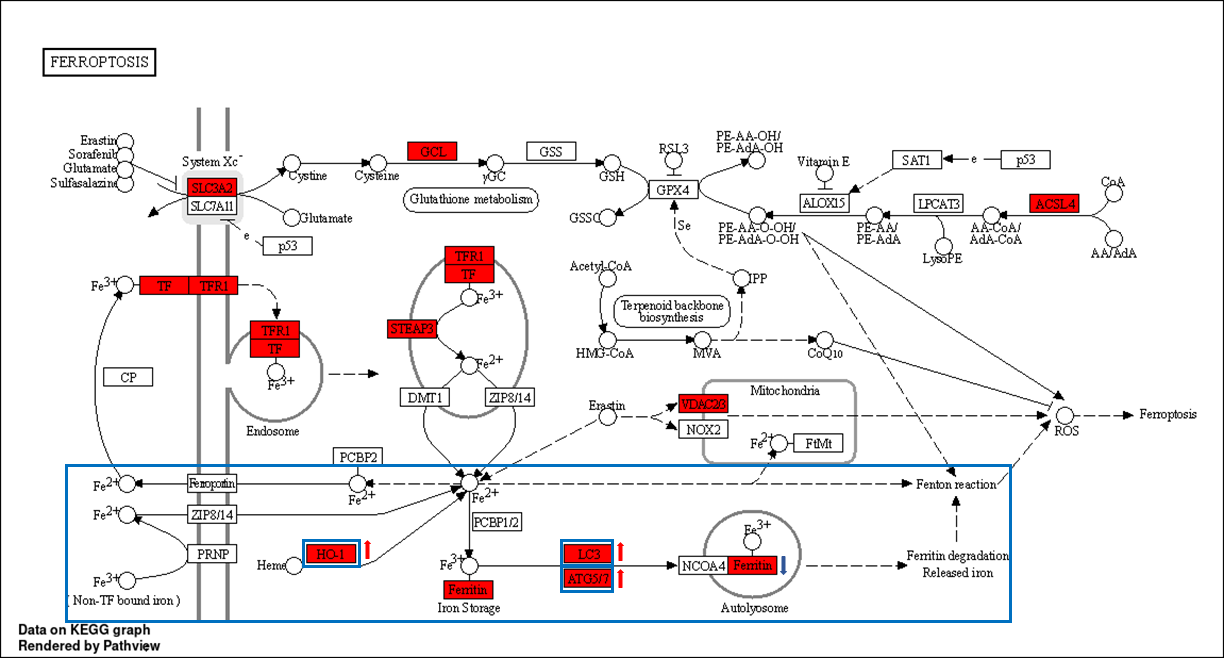
Figure S1. Ferroptosis related KEGG signaling pathway.


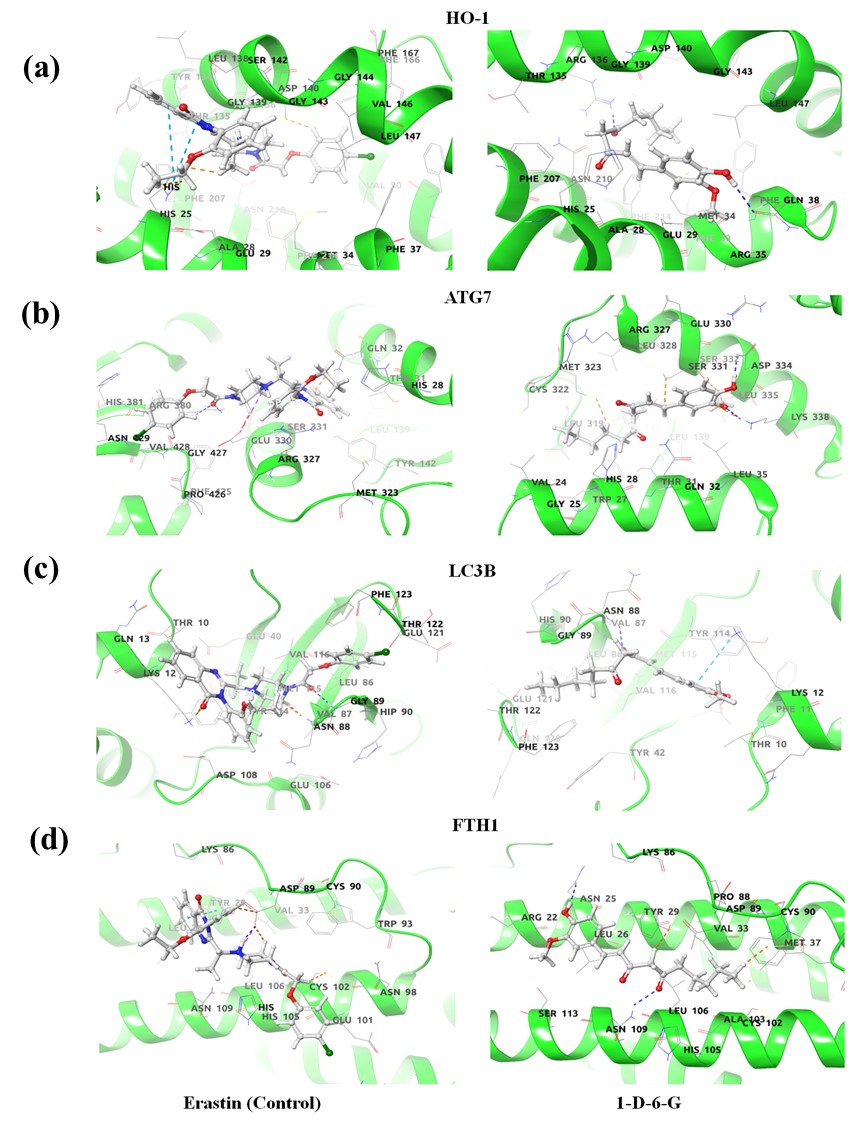


Figure S2. Molecular docking of 1-D-6-G and Erastin with Ferroptosis related proteins. Interactions of 1-D-6-G and Erastin with (a) HO-1, (b) ATG7, (c) LC3B, and (d) FTH1.

Supplementary Table S1: Primer sequences used for qRT-PCR analysis

| Primer | Sequence | |
| --- | --- | --- |
| GAPDH | Forward | 5’-ACCACAGTCCATGCCATCAC-3’ |
|  | Reverse | 5’-CCA CCA CCC TGT TGC TGT AG-3’ |
| FTH1 | Forward | 5'-GCTCTACGCCTCCTACGTTTACCT-3' |
|  | Reverse | 5'-TGTCTCCCAGGGTGTGCTTGTCAA-3' |
| LC3B | Forward | 5'-ATAATTAGAAGGCGCTTACAGCTC-3' |
|  | Reverse | 5'-TGGCAGGTTCTCTTCTCTAGATCT-3' |
| HO-1 | Forward | 5'-GAATGCTGAGTTCATGAGGAACTT-3' |
|  | Reverse | 5'-GCCTTGCGGTGCAGCTCT-3' |
| ATG7 | Forward | 5'-TAG AGC TGC GGA CTG CCT TCA-3' |
|  | Reverse | 5'-GGT CTT CAG CTT CTC ACC CAG-3' |
